# Routine Generative AI Use and Acceptance of Medical AI: A Cross-Sectional Survey of Healthcare Workers and the Public in Japan

**DOI:** 10.7759/cureus.110118

**Published:** 2026-06-02

**Authors:** Toshiharu Mitsuhashi

**Affiliations:** 1 Center for Innovative Clinical Medicine, Medical Development Field, Okayama University, Okayama, JPN

**Keywords:** generative ai, healthcare workers, medical ai, non-healthcare workers, technology acceptance, web-based survey

## Abstract

Background

Acceptance of medical artificial intelligence (AI) is critical for successful implementation, yet its acceptance may vary by clinical application and stakeholder group. Whether routine use of generative AI (GAI) is associated with acceptance of medical AI remains unclear.

Methods

A cross-sectional web survey was conducted in Japan in November 2025 using web-panel convenience sampling from an online research panel. The small analytic sample comprised 200 participants aged 20-69 years, including 100 healthcare workers and 100 non-healthcare workers. GAI use was assessed using two five-point items (GAI use at work and in daily life) and dichotomized as at least monthly use in either daily life or work versus less than monthly use in both settings, based on prespecified criteria. Acceptance of medical AI was measured for five scenario-based applications - AI-assisted imaging interpretation, AI-based health risk prediction, AI-based treatment recommendations, AI-enabled triage guidance, and AI-assisted robotic surgery - using a four-point acceptability scale; responses were dichotomized as acceptable versus not acceptable. Calibration weights were constructed to approximate Japanese internet users by raking on sex-by-age group and household income, normalized to the analytic sample size, and truncated at prespecified bounds. Adjusted prevalence ratios (aPRs) were estimated using modified Poisson regression with robust standard errors, stratified by occupation; covariates were selected a priori using a modified disjunctive cause criterion. This study was exploratory; therefore, adjustments for multiple comparisons were not performed.

Results

Among healthcare workers, the lower-use group had a higher prevalence of non-acceptance of AI-based health risk prediction (aPR 6.980, 95% CI 1.412-34.506) and AI-based treatment recommendations (aPR 4.364, 95% CI 1.331-14.305), whereas estimates for imaging interpretation, triage guidance, and robotic surgery remained statistically uncertain. Among non-healthcare workers, the lower-use group had a higher prevalence of non-acceptance of AI-assisted imaging interpretation (aPR 13.906, 95% CI 1.624-119.052), AI-based health risk prediction (aPR 4.861, 95% CI 1.133-20.862), AI-based treatment recommendations (aPR 6.288, 95% CI 1.386-28.530), and AI-assisted robotic surgery (aPR 6.173, 95% CI 1.435-26.561); the adjusted estimate for AI-enabled triage guidance was not statistically supported (aPR 1.840, 95% CI 0.521-6.505). Several estimates had wide confidence intervals, indicating substantial imprecision.

Conclusions

In this small exploratory web-panel convenience sample, lower-frequency GAI use was associated with higher non-acceptance of several medical AI applications. Associations were observed across more applications among non-healthcare workers than among healthcare workers. Because the study was cross-sectional, used convenience sampling, and included imprecise estimates with wide confidence intervals, the findings should not be interpreted causally. Larger studies using longitudinal or interventional designs are needed to clarify temporality and mechanisms.

## Introduction

Medical artificial intelligence (medical AI) is increasingly discussed as a practical component of healthcare, from decision support to patient-facing services. In this study, medical AI refers to AI-based software or services used in healthcare or health-related contexts to support information provision, screening or triage, diagnosis, risk prediction, treatment or lifestyle recommendations, documentation, or patient-clinician communication. The term does not imply full replacement of clinicians; rather, it includes a broad range of applications that differ in clinical proximity, autonomy, and potential consequences for patients. Implementation depends not only on technical performance but also on whether intended users consider a system acceptable and trustworthy. Prior work has emphasized that concerns about privacy, accountability, and fairness can impede adoption even when potential benefits are acknowledged [1]. Furthermore, attitudes towards technology and its use also influence the acceptance of medical AI [2].

Theoretical models of technology adoption provide a rationale for examining prior GAI use as a possible correlate of medical AI acceptance. The Technology Acceptance Model proposes that perceived usefulness and perceived ease of use are central determinants of technology acceptance [3]. Diffusion of Innovations theory further suggests that adoption is shaped by perceived attributes of an innovation, including relative advantage, compatibility, complexity, trialability, and observability [4]. In parallel, the trust in automation literature emphasizes that trust guides reliance on automated systems, particularly when systems are complex and their internal operation cannot be fully understood by users [5]. From these perspectives, routine experience with GAI may increase familiarity with AI systems, make AI more observable and trialable, enhance perceived ease of use and digital confidence, and reduce uncertainty about AI outputs. These considerations provide a theoretical rationale for examining the association between routine GAI use and acceptance of medical AI. Conversely, negative experiences with GAI may reduce trust or heighten perceived risks.

Importantly, “medical AI” covers heterogeneous applications, and acceptance can differ across use cases. Survey and preference-elicitation studies suggest that intention to use, trust, and willingness to engage with AI vary depending on the clinical task, proximity to the patient, and perceived consequences of errors or trade-offs (e.g., data sharing, service attributes) [2,6]. This heterogeneity implies that a single global attitude question may obscure meaningful variation in acceptance across contexts.

Acceptance may also differ between stakeholder groups. Evidence from studies on AI-related attitudes and readiness indicates that professional context, digital competence, and perceived usefulness are associated with the intention to adopt AI tools and that patterns can differ between healthcare workers and patients or the general public [6-8]. These observations motivate explicit comparisons between healthcare workers and non-healthcare workers when evaluating factors linked to acceptance.

A further timely question is whether experience with generative artificial intelligence (GAI, e.g., tools represented by ChatGPT) spills over into broader attitudes toward medical AI. GAI is now encountered in routine information work and has begun to be discussed in clinical settings; a recent longitudinal survey also reported that exposure to ChatGPT was associated with reduced uncertainty and more positive perceptions of AI in healthcare among individuals who had been uncertain at baseline [9]. Physicians have also been surveyed regarding GAI integration and its potential implications for patient care [10]. Despite this emerging attention, empirical evidence directly linking routine GAI use to acceptance of distinct medical AI applications remains limited, particularly in designs that explicitly contrast healthcare workers with the public and differentiate application domains.

This exploratory cross-sectional web survey, therefore, aimed to assess whether routine GAI use was associated with acceptance of medical AI across five application scenarios. A second aim was to examine whether the pattern of association differed between healthcare workers and non-healthcare workers. Because the study was designed as a hypothesis-generating study, emphasis was placed on estimating the direction and uncertainty of associations rather than on making causal claims.

## Materials and methods

Study design and setting

A cross-sectional study was conducted using a web-based survey of registered panelists at Rakuten Insight Inc., Tokyo, Japan. This manuscript was prepared in accordance with the Strengthening the Reporting of Observational Studies in Epidemiology (STROBE) statement for observational studies [11] and Checklist for Reporting Results of Internet E-Surveys (CHERRIES) [12].

A web-based questionnaire was fielded from November 14 to 17, 2025, and respondents received Rakuten Points as an incentive; the number of points awarded is confidential information held by Rakuten Insight Inc. The dataset provided for analysis contained no information that could be used to identify individuals. Questionnaire items were developed by the researcher, while the user interface and web-based survey settings were implemented by Rakuten Insight Inc. An English translation of the web-based questionnaire is provided in Appendix 1. The questionnaire was not pretested before fielding. Specifically, no pilot survey, cognitive interviewing, or formal validation study was conducted to assess whether respondents interpreted the items on GAI use and medical AI acceptance as intended. Potential participants were notified via email and/or a smartphone application. No adaptive questioning (skip logic) was used. The survey was administered online, with one questionnaire item displayed per page. It comprised four screening items (including an item indicating consent to participate) and a 13-page main questionnaire. All items were mandatory, and respondents could not proceed to the next page if any were unanswered; however, they could review and change their answers before final submission. The survey system was configured to prevent multiple submissions from the same individual.

Participants and subgroups

Eligibility criteria were as follows: (1) Rakuten Insight members aged 20 or over, (2) not a student, and (3) having worked in the current occupation for at least three years. Exclusion criteria were (1) inability to provide informed consent and (2) engagement in work related to GAI. Screening questions were used so that only eligible participants proceeded to the main questionnaire.

Because this study was exploratory and hypothesis-generating, a power-based sample size calculation was not performed. The sample size was determined based on budgetary and operational constraints. The planned sample size was 200 participants, with 100 healthcare workers and 100 non-healthcare workers, to enable exploratory occupation-specific analyses. This sample size was not intended to provide stable estimates for all subgroup-specific and scenario-specific models.

The target population was defined as internet users in Japan. However, because a commercial web-survey panel was used, probability-based random sampling was not feasible. Among candidates who received the invitation to the web-based questionnaire, eligible participants were enrolled on a first-come, first-served basis. Data collection was terminated when the occupation-specific quotas were reached (100 healthcare workers and 100 non-healthcare workers), yielding a total of 200 participants.

This sampling procedure may have introduced selection bias. In particular, participants were limited to registered web-panel members who responded to the survey invitation before the quotas were filled. Therefore, the sample may overrepresent individuals with higher digital literacy, greater willingness to participate in online surveys, or a stronger interest in technology or health-related topics. These features should be considered when interpreting the generalizability of the findings.

Variable definition

Exposure: GAI Use

Respondents were asked to rate their “GAI use at work” and “GAI use in daily life (outside work)” on a five-point scale (almost daily/several times a week/several times a month/rarely/never). Respondents who reported using GAI several times a month or more in either setting were classified into the higher-use group, whereas respondents who reported “rarely” or “never” in both settings were classified into the lower-use group.

Outcome: Acceptance of Medical AI

Acceptance of medical AI was assessed using a single question: “If you were a patient, to what extent would you accept the use of such an AI in your own surgery?” Responses were recorded on a four-point scale (“acceptable,” “somewhat acceptable,” “somewhat unacceptable,” and “unacceptable”). Participants who responded “acceptable” or “somewhat acceptable” were classified as demonstrating “acceptance,” and all other responses were classified as “non-acceptance.”

The outcome was assessed for the following five types of medical AI. These five items were presented on screen in a randomized order: (i) AI-assisted imaging interpretation; (ii) AI-based health risk prediction; (iii) AI-based treatment recommendations; (iv) AI-enabled triage guidance; and (v) AI-assisted robotic surgery.

The same question stem was used across all five scenarios, and only the scenario description preceding the item differed. Table 1 summarizes the explanatory text shown to respondents, along with the medical-AI characteristics and question intents (i.e., domain, proximity to the patient, and severity) prespecified by the investigators (which were not shown to respondents).

**Table 1:** Questionnaire scenarios and investigator-assigned characteristics of the five medical AI applications The second column shows the scenario descriptions presented to respondents. The remaining columns summarize conceptual characteristics assigned a priori by the investigators (domain involved, proximity to the patient, and severity) and were not shown to respondents. AI, artificial intelligence; CT, computed tomography

Medical AI category	Question statements in the questionnaire	Domain involved	Proximity to the patient	Severity
AI-assisted imaging interpretation	AI analyzes your X-ray or CT images taken by a physician, detects areas suspicious for disease (e.g., cancer or fractures), and reports them to the physician. The final diagnosis is made by the physician with reference to the AI output.	Diagnosis (support)	Distant (a tool for physicians)	Low to moderate
AI-based health risk prediction	AI analyzes daily health data measured by devices such as a smartwatch that you wear (e.g., heart rate) and predicts disease risk, for example, “You are at high risk of developing a dangerous arrhythmia within a few hours.” AI then issues an alert to you and/or your family.	Prevention and monitoring	Close (alerts the individual)	Moderate to high
AI-based treatment recommendations	AI comprehensively analyzes your lifestyle, past medical history, and the latest medical literature, and proposes multiple treatment options (e.g., medication type and use) that are expected to be most effective for you to the physician. The final decision on which treatment to choose is made through consultation between the physician and you.	Treatment (recommendation)	Distant (a tool for physicians)	High
AI-enabled triage guidance	You interact with an AI chatbot on a hospital website. Based on the symptoms you enter, AI automatically asks follow-up questions to organize the information and provides guidance on the appropriate department and urgency, for example, “High urgency - go to the emergency department” or “Please make an appointment with internal medicine.”	Communication	Close (direct interaction)	Low
AI-assisted robotic surgery	When you undergo surgery, the physician-operated surgical robot is equipped with AI. AI supports precise maneuvers that are difficult for humans alone, such as stabilizing the surgeon’s hand movements and helping prevent deviation from a preplanned surgical range. The overall procedure is performed and supervised by a human physician.	Treatment (physical intervention)	Very close (physical contact)	Very high

The acceptance items were investigator-developed single-item measures for each medical AI scenario. They were intended to assess stated acceptance of specific hypothetical medical AI applications and were not based on a validated psychometric scale.

Covariates

Covariates were selected a priori based on modified disjunctive cause criterion principles [13]. Variables were included if they were considered plausible common causes of both GAI use and acceptance of medical AI, or strong predictors of either the exposure or the outcome. Sex, age, education, and household annual income were included to account for demographic and socioeconomic differences. Interest in new technologies was included because it may influence both GAI use and openness toward medical AI. The presence of chronic disease was included because personal experience with healthcare may affect attitudes toward medical AI.

To adjust for confounding, the following covariates were used in the adjusted model: sex (dichotomous variable), age (continuous variable, including a quadratic term), interest in new technologies (dichotomous variable), education (five-level categorical variable), household annual income (three-level categorical variable), and presence of chronic disease (dichotomous variable). The same adjustment set was used in both occupational subgroups to maintain comparability and to avoid data-driven covariate selection in this small exploratory study. Occupation itself was not included as a covariate in the primary subgroup-specific models because the primary analyses were stratified by healthcare worker status.

Due to budget constraints for the web survey, the number of variables that could be collected was limited. Consequently, variables likely to have a stronger influence were prioritized for collection; it is possible that not all variables requiring adjustment were obtained.

Gender, age, and annual household income were obtained from the registration information of the web research company. Interest in new technology was defined as “interested” when respondents answered “very interested” or “somewhat interested” to the question “Are you usually interested in information about new technologies such as smartphones, apps, or web services?”, using a five-point scale (very/somewhat/neutral/not very/not at all). Education was measured using a five-point scale (Junior High School/High School/Some College/University/Graduate School). Household annual income was categorized into three levels based on web research registration data (6 million JPY or less/over 6 million JPY to 10 million JPY/over 10 million JPY). Presence of chronic disease was determined by responses to the question “Do you currently have a chronic disease (such as hypertension, diabetes, asthma, and so on) requiring regular hospital visits or treatment?” with answers of “Yes” or “No.”

Addressing bias

Because this was a cross-sectional web survey, several sources of bias were anticipated, including confounding, selection bias related to participation (which can be conceptualized as conditioning on a collider [14]), and measurement error. Confounding was addressed through covariate adjustment in multivariable models. Selection bias due to non-probability panel sampling was partially mitigated through calibration weighting to align the sample with external benchmarks for Japanese internet users. Residual selection bias may remain. Measurement error could not be eliminated because exposure and outcome were self-reported; if misclassification was largely non-differential, associations would generally be biased toward the null.

Statistical analysis

All statistical analyses were performed using StataNow 19.5/MP8 (StataCorp LLC, College Station, TX, USA). A p-value of <0.05 was regarded as nominal statistical support, but not as a basis for confirmatory inference. This study was exploratory; therefore, adjustments for multiple comparisons were not performed [15]. Therefore, the findings were interpreted based on the direction, magnitude, precision, and consistency of the estimates, together with nominal p-values, rather than on p-values alone.

Descriptive Statistics

To describe the collected samples, the distributions of covariates and outcomes were summarized for the healthcare worker and non-healthcare worker subgroups, stratified by exposure status. Continuous variables were summarized by mean and standard deviation, while categorical variables were summarized by frequency and proportion. In accordance with the STROBE statement, no statistical tests were performed on the descriptive statistics.

Primary Analysis: Estimation of Association by Subgroup

The sample in this study was not proportionate to the population ratio of healthcare workers to non-healthcare workers. Consequently, there are limitations in estimating overall outcome prevalence and in extrapolating results for the general population. Therefore, the primary analysis was conducted by occupational subgroup to assess associations within each stratum.

Calibration weights were constructed to improve the population representativeness of the web-based survey sample [16]. Japanese internet users were defined as the target population. External benchmarks were obtained from public tabulations of the Ministry of Internal Affairs and Communications’ Communications Usage Trend Survey (Household Members; e-Stat [17]), restricted to ages 20-69 years. Iterative proportional fitting (raking) was used to calibrate the weights so that the weighted marginal distributions of sex-by-age group and household income (three categories) matched the benchmarks. The calibrated weights were normalized to sum to the analytic sample size and truncated at prespecified bounds (lower bound: 0.1; upper bound: 10). Weight variability was assessed using Kish’s effective sample size (ESS).

In this cross-sectional study, the prevalence ratio (PR) was estimated as the measure of association for dichotomous outcomes. Modified Poisson regression (Poisson regression with robust standard errors) was used to estimate PRs [18]. For each outcome, two models were fitted separately in the healthcare worker and non-healthcare worker subgroups. The crude model included only the exposure variable. The adjusted model included the exposure variable and all covariates. In the primary models, the dichotomous outcome was coded as non-acceptance (=1) versus acceptance (=0), and the exposure contrast was the lower-use group versus the higher-use group. Therefore, PRs greater than 1 indicated a higher prevalence of non-acceptance in the lower-use group. Estimation was performed using Stata’s poisson command with robust standard errors to obtain point estimates and 95% confidence intervals (CIs).

As a supplementary absolute-scale measure, crude prevalence differences (PDs) and adjusted prevalence differences (aPDs) were calculated as marginal differences in predicted prevalence between the lower-use and higher-use groups, using the same crude and adjusted model specifications. Delta-method standard errors were used to estimate 95% CIs and p-values when available. If standard errors could not be estimated because of sparse non-acceptance events, only the point estimate was shown.

Sensitivity Analysis: E-values

As a sensitivity analysis for unmeasured confounding, E-values were calculated as proposed by VanderWeele and Ding [19]. The E-value summarizes, on a risk ratio scale, the minimum strength of association that an unmeasured confounder would need to have with both the exposure and the outcome (conditional on measured covariates) to fully explain away an observed PR. E-values were reported for each PR point estimate. When the 95% confidence interval excluded the null (PR = 1), the E-value for the confidence limit closest to the null was also reported. E-values were computed using the Stata user-written command `evalue` [20].

Sensitivity Analysis: Four-Level GAI Use Frequency

To assess whether the dichotomous exposure definition oversimplified variation in GAI use intensity, an additional sensitivity analysis was conducted using a four-level GAI use frequency variable. GAI use in daily life, and GAI use at work were assessed using the same five response categories: almost daily, several times a week, several times a month, rarely, and never. For each participant, the highest reported frequency across daily life and work was used to define overall GAI use frequency.

Because few participants reported almost daily use, almost daily use and use several times a week were combined. The final categories were several times a week or more, several times a month, rarely, and never. This variable was modeled as a categorical exposure variable in the modified Poisson regression models, with several times a week or more as the reference category.

Supplemental Analysis: Effect Modification (Multiplicative Interaction Term)

As a supplemental analysis, effect modification by occupation on the multiplicative scale was assessed by fitting a model in the full sample that included GAI use group, occupation (healthcare worker vs non-healthcare worker), their product term (GAI use × occupation), and all covariates. The product term PR was interpreted as the ratio of the PR for the lower-use group versus the higher-use group among non-healthcare workers to the corresponding PR among healthcare workers. Subgroup-specific PRs were treated as the primary presentation of heterogeneity, and the product term was reported as supplemental information.

Handling Missing Values

No missing values were present in the variables used for analysis due to the web-survey design.

Data and Code Availability

The analysis code and non-identifiable raw data are publicly available at Zenodo (https://doi.org/10.5281/zenodo.20411901).

Ethical approval

This study was ethically approved by the Ethics Committee of the Okayama University Graduate School of Medicine, Dentistry, and Pharmaceutical Sciences, and Okayama University Hospital (approval date: October 17, 2025; approval number: K2510-011). The purpose and methods of the research were appropriately described to potential participants on the recruitment web page. After this description, participants provided informed consent. They were free to refuse to participate for any reason.

Use of large language models

Large language models (ChatGPT 5.2 Thinking, OpenAI, San Francisco, CA, USA, and Gemini 3 Pro, Google DeepMind, London, United Kingdom) were used for English proofreading and to flag potentially illogical statements and missing references.

## Results

Participants

A total of 200 respondents who completed the web-based questionnaire were included in the analysis, comprising 100 healthcare workers and 100 non-healthcare workers. No missing data were observed. Because the number of invitations distributed for the web-based questionnaire was unknown, the response rate could not be calculated. Kish’s effective sample size was 61.90 among healthcare workers and 55.82 among non-healthcare workers.

Descriptive statistics

Descriptive characteristics are shown in Table 2. The analytic sample consisted of 100 healthcare workers, including 32 in the higher-use group and 68 in the lower-use group, and 100 non-healthcare workers, including 37 in the higher-use group and 63 in the lower-use group. Across all five outcomes, non-acceptance events were sparse in the higher-use groups. The number of non-acceptance events ranged from 2 to 4 per outcome among healthcare workers with higher GAI use and from 1 to 3 per outcome among non-healthcare workers with higher GAI use. The corresponding ranges in the lower-use groups were 19 to 24 and 17 to 20 events per outcome, respectively.

**Table 2:** Participant characteristics by occupation and generative AI use group Data are presented as n (%) for categorical variables and mean (SD) for continuous variables. Percentages are column percentages. The higher-use group comprised participants reporting at least monthly GAI use in either daily life or work, whereas the lower-use group comprised participants reporting less than monthly use in both settings. This table is presented for descriptive purposes to summarize participant characteristics across groups. Consistent with STROBE-oriented reporting for descriptive tables, no p-values or hypothesis test statistics are presented. GAI, generative artificial intelligence; SD, standard deviation

	Healthcare worker		Non-healthcare worker	
	Higher-use group	Lower-use group	Higher-use group	Lower-use group
	32 (32.0%)	68 (68.0%)	37 (37.0%)	63 (63.0%)
Socio-demographic Variables				
Sex				
Female	12 (37.5%)	34 (50.0%)	15 (40.5%)	16 (25.4%)
Male	20 (62.5%)	34 (50.0%)	22 (59.5%)	47 (74.6%)
Age	46.12 (9.66)	51.13 (9.79)	51.08 (10.16)	54.94 (7.50)
Education				
Junior high school	0 (0.0%)	0 (0.0%)	0 (0.0%)	1 (1.6%)
High school	1 (3.1%)	7 (10.3%)	7 (18.9%)	19 (30.2%)
Some college	10 (31.2%)	26 (38.2%)	8 (21.6%)	15 (23.8%)
University	16 (50.0%)	33 (48.5%)	19 (51.4%)	25 (39.7%)
Graduate school	5 (15.6%)	2 (2.9%)	3 (8.1%)	3 (4.8%)
Annual income (JPY)				
6 million or less	7 (21.9%)	31 (45.6%)	16 (43.2%)	34 (54.0%)
6 to 10 million	10 (31.2%)	18 (26.5%)	13 (35.1%)	20 (31.7%)
More than 10 million	15 (46.9%)	19 (27.9%)	8 (21.6%)	9 (14.3%)
Chronic disease	8 (25.0%)	14 (20.6%)	14 (37.8%)	22 (34.9%)
Interest in new technologies	20 (62.5%)	18 (26.5%)	26 (70.3%)	20 (31.7%)
Non-acceptance of each Medical AI				
AI-assisted imaging interpretation	4 (12.5%)	19 (27.9%)	1 (2.7%)	17 (27.0%)
AI-based health risk prediction	2 (6.2%)	19 (27.9%)	3 (8.1%)	19 (30.2%)
AI-based treatment recommendations	2 (6.2%)	23 (33.8%)	3 (8.1%)	17 (27.0%)
AI-enabled triage guidance	3 (9.4%)	24 (35.3%)	3 (8.1%)	17 (27.0%)
AI-assisted robotic surgery	3 (9.4%)	22 (32.4%)	3 (8.1%)	20 (31.7%)

Main results

**Figure 1:**
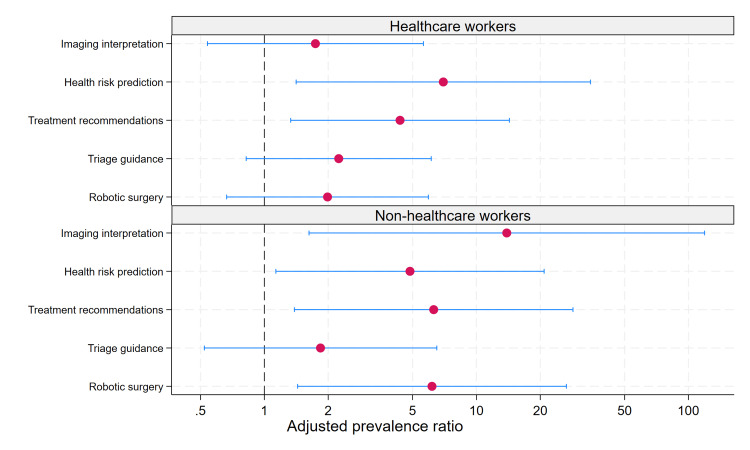
Adjusted prevalence ratios for non-acceptance of medical AI applications according to lower versus higher generative AI use Adjusted prevalence ratios were estimated using modified Poisson regression with robust standard errors. The reference group was participants with higher generative AI use. Points indicate adjusted prevalence ratios, and horizontal bars indicate 95% confidence intervals. The vertical line indicates a prevalence ratio of 1.0. GAI, generative artificial intelligence; AI, artificial intelligence

The adjusted associations between lower GAI use and non-acceptance of each medical AI application are summarized in Table 3 and Figure 1. Among healthcare workers, the adjusted prevalence ratios (aPRs) for AI-based health risk prediction and AI-based treatment recommendations had confidence intervals that did not include 1.0. For the remaining outcomes, the confidence intervals included 1.0, indicating substantial statistical uncertainty.

**Table 3:** Associations of generative AI use group with non-acceptance of each medical AI application by occupational subgroup: prevalence ratios and prevalence differences Data are presented as PRs or aPRs and PDs or aPDs with 95% confidence intervals for the prevalence of non-acceptance of each medical AI application in the lower-use group compared with the higher-use group within each occupational subgroup. For PDs and aPDs, positive values indicate a higher absolute prevalence of non-acceptance in the lower-use group. The higher-use group comprised participants reporting at least monthly GAI use in either daily life or work, whereas the lower-use group comprised participants reporting less than monthly use in both settings. Crude and adjusted PRs or aPRs were obtained using modified Poisson regression with robust standard errors. Crude and adjusted PDs or aPDs were calculated as marginal differences in predicted prevalence, and standard errors, 95% confidence intervals, and p-values were estimated using the delta method. When delta-method standard errors and confidence intervals could not be estimated because of sparse non-acceptance events, only the point estimate is shown, and the confidence interval and p-value are indicated as N/A. P-values were derived from Wald tests and were not adjusted for multiple comparisons. A p-value of <0.05 was regarded as nominal statistical support. E-values are presented only for PRs and aPRs, including the point estimate and the confidence limit closest to the null. E-values were not calculated for PDs or aPDs because an E-value is not uniquely determined from a prevalence difference alone. PR, prevalence ratio; aPR, adjusted prevalence ratio; PD, prevalence difference; aPD, adjusted prevalence difference; CI, confidence interval; AI, artificial intelligence; GAI, generative artificial intelligence; N/A, not available

Subgroup	Outcome	Model	PR/aPR (95% CI)	p-value	E-value (PR)	E-value (95% CI)	PD/aPD (95% CI)	p-value
Healthcare worker	AI-assisted imaging interpretation	Crude	2.271 (0.682, 7.560)	0.182	3.969	1	0.160 (-0.030, 0.349)	0.099
		Adjusted	1.741 (0.539, 5.623)	0.354	2.876	1	0.112 (-0.098, 0.321)	0.296
	AI-based health risk prediction	Crude	11.187 (2.323, 53.886)	0.003	21.863	4.075	0.310 (0.158, 0.462)	<0.001
		Adjusted	6.980 (1.412, 34.506)	0.017	13.440	2.174	0.254 (0.128, 0.380)	<0.001
	AI-based treatment recommendations	Crude	4.578 (0.825, 25.398)	0.082	8.626	1.000	0.264 (0.082, 0.446)	0.004
		Adjusted	4.364 (1.331, 14.305)	0.015	8.196	1.996	0.256 (0.115, 0.398)	<0.001
	AI-enabled triage guidance	Crude	3.295 (0.880, 12.338)	0.077	6.045	1.000	0.247 (0.054, 0.439)	0.012
		Adjusted	2.243 (0.821, 6.124)	0.115	3.912	1.000	0.175 (0.003, 0.347)	0.046
	AI-assisted robotic surgery	Crude	3.199 (0.907, 11.276)	0.071	5.850	1.000	0.255 (0.054, 0.456)	0.013
		Adjusted	1.986 (0.664, 5.934)	0.219	3.384	1.000	0.160 (-0.050, 0.371)	0.135
Non-healthcare worker	AI-assisted imaging interpretation	Crude	28.248 (3.642, 219.064)	0.001	55.991	6.745	0.319 (0.167, 0.471)	<0.001
		Adjusted	13.906 (1.624, 119.052)	0.016	27.303	2.631	0.237 (N/A)	N/A
	AI-based health risk prediction	Crude	8.958 (2.255, 35.592)	0.002	17.402	3.937	0.336 (0.173, 0.500)	<0.001
		Adjusted	4.861 (1.133, 20.862)	0.033	9.194	1.521	0.239 (N/A)	N/A
	AI-based treatment recommendations	Crude	10.701 (2.952, 38.789)	0.000	20.890	5.353	0.311 (0.154, 0.469)	<0.001
		Adjusted	6.288 (1.386, 28.530)	0.017	12.055	2.118	0.236 (N/A)	N/A
	AI-enabled triage guidance	Crude	2.045 (0.478, 8.749)	0.335	3.507	1.000	0.154 (-0.094, 0.402)	0.224
		Adjusted	1.840 (0.521, 6.505)	0.344	3.083	1.000	0.131 (N/A)	N/A
	AI-assisted robotic surgery	Crude	8.518 (2.023, 35.874)	0.003	16.521	3.461	0.324 (0.160, 0.488)	<0.001
		Adjusted	6.173 (1.435, 26.561)	0.014	11.825	2.225	0.270 (-0.248, 0.788)	0.308

Among non-healthcare workers, the aPRs for AI-assisted imaging interpretation, AI-based health risk prediction, AI-based treatment recommendations, and AI-assisted robotic surgery had confidence intervals that did not include 1.0. However, the confidence intervals were wide, particularly for AI-assisted imaging interpretation, and the estimates should therefore be interpreted with caution. For AI-enabled triage guidance, the confidence interval included 1.0.

On the absolute scale, aPDs are also shown in Table 3. Among healthcare workers, the aPDs ranged from 0.112 to 0.256, with varying precision across outcomes. Among non-healthcare workers, aPD point estimates ranged from 0.131 to 0.270. However, confidence intervals and p-values could not be estimated for several non-healthcare outcomes because of sparse non-acceptance events; these absolute-scale estimates are therefore presented descriptively.

Sensitivity Analysis: E-values

E-values are shown in Table 3. For aPRs with confidence intervals that did not include 1.0, the E-values for the point estimates ranged from 8.196 to 27.303. The E-values for the confidence limit closest to the null ranged from 1.521 to 2.631.

Sensitivity Analysis: Four-Level GAI Use Frequency

The sensitivity analysis using the four-level GAI use frequency is shown in Table 4. Several estimates had wide confidence intervals, and some estimates were unstable, particularly in categories with sparse outcome events. Therefore, this analysis was interpreted as exploratory and was not used to infer a dose-response pattern.

**Table 4:** Sensitivity analysis of associations between four-level generative AI use frequency and non-acceptance of each medical AI application by occupational subgroup Data are presented as PRs or aPRs with 95% confidence intervals for the prevalence of non-acceptance of each medical AI application by four-level GAI use frequency within each occupational subgroup. The four-level GAI use frequency variable was created using the highest reported frequency of GAI use across daily life and work. Because few participants reported almost daily GAI use, almost daily use and use several times a week were combined. The final categories were several times a week or more, several times a month, rarely, and never. The reference category was several times a week or more. Crude and adjusted estimates were obtained using modified Poisson regression with robust standard errors. p-values were derived from Wald tests. Statistical significance was defined as p < 0.05. E-values are presented for the point estimate and for the confidence limit closest to the null. PR, prevalence ratio; aPR, adjusted prevalence ratio; CI, confidence interval; AI, artificial intelligence; GAI, generative artificial intelligence

Subgroup	Outcome	Model	Exposure level	PR/aPR (95% CI)	p-value	E-value (PR)	E-value (95% CI)
Healthcare worker	AI-assisted imaging interpretation	Crude	Several times a month	1.696 (0.187, 15.353)	0.638	2.783	1.000
			Rarely	1.637 (0.208, 12.885)	0.640	2.658	1.000
			Never	3.708 (0.530, 25.933)	0.187	6.876	1.000
		Adjusted	Several times a month	2.201 (0.395, 12.276)	0.368	3.827	1.000
			Rarely	1.647 (0.331, 8.198)	0.543	2.678	1.000
			Never	3.584 (0.912, 14.086)	0.068	6.628	1.000
	AI-based health risk prediction	Crude	Several times a month	9.1 × 10^6^ (1.7 × 10^6^, 4.8 × 10^7^)	<0.001	18000000	3500000
			Rarely	3.9 × 10^7^ (1.2 × 10^7^, 9.5 × 10^7^)	<0.001	68000000	24000000
			Never	3.9 × 10^7^ (1.4 × 10^7^, 1.0 × 10^8^)	<0.001	77000000	28000000
		Adjusted	Several times a month	2.8 × 10^7^ (4.1 × 10^6^, 1.9 × 10^8^)	<0.001	56000000	8200000
			Rarely	6.7 × 10^7^ (1.7 × 10^7^, 2.6 × 10^8^)	<0.001	130000000	35000000
			Never	2.8 × 10^7^ (6.6 × 10^6^, 1.2 × 10^8^)	<0.001	57000000	13000000
	AI-based treatment recommendations	Crude	Several times a month	0.263 (0.017, 4.089)	0.340	7.053	1.000
			Rarely	2.427 (0.326, 18.088)	0.387	4.287	1.000
			Never	4.040 (0.583, 28.016)	0.158	7.544	1.000
		Adjusted	Several times a month	0.347 (0.029, 4.147)	0.403	5.205	1.000
			Rarely	2.663 (0.621, 11.428)	0.188	4.767	1.000
			Never	3.929 (1.044, 14.779)	0.043	7.321	1.260
	AI-enabled triage guidance	Crude	Several times a month	0.717 (0.061, 8.501)	0.792	2.136	1.000
			Rarely	2.139 (0.370, 12.364)	0.396	3.700	1.000
			Never	3.551 (0.668, 18.868)	0.137	6.561	1.000
		Adjusted	Several times a month	0.741 (0.119, 4.596)	0.747	2.037	1.000
			Rarely	1.585 (0.398, 6.320)	0.514	2.549	1.000
			Never	2.354 (0.720, 7.701)	0.157	4.140	1.000
	AI-assisted robotic surgery	Crude	Several times a month	1.433 (0.144, 14.252)	0.759	2.220	1.000
			Rarely	2.995 (0.410, 21.867)	0.279	5.440	1.000
			Never	4.204 (0.606, 29.176)	0.146	7.873	1.000
		Adjusted	Several times a month	2.693 (0.432, 16.778)	0.289	4.828	1.000
			Rarely	3.668 (0.840, 16.008)	0.084	6.796	1.000
			Never	2.624 (0.671, 10.266)	0.166	4.688	1.000
Non-healthcare worker	AI-assisted imaging interpretation	Crude	Several times a month	0.000 (0.000, 0.000)	0.000	4700000	1.000
			Rarely	12.582 (1.375, 115.157)	0.025	24.654	2.092
			Never	22.962 (2.853, 184.830)	0.003	45.419	5.152
		Adjusted	Several times a month	0.000 (0.000, 0.000)	0.000	5700000	1.000
			Rarely	3.219 (0.307, 33.762)	0.330	5.891	1.000
			Never	8.881 (0.844, 93.476)	0.069	17.246	1.000
	AI-based health risk prediction	Crude	Several times a month	1.337 (0.113, 15.798)	0.818	2.009	1.000
			Rarely	8.170 (0.992, 67.289)	0.051	15.824	1.000
			Never	11.327 (1.469, 87.370)	0.020	22.143	2.298
		Adjusted	Several times a month	0.884 (0.074, 10.565)	0.922	1.517	1.000
			Rarely	3.427 (0.407, 28.831)	0.257	6.311	1.000
			Never	5.487 (0.639, 47.086)	0.121	10.449	1.000
	AI-based treatment recommendations	Crude	Several times a month	3.337 (0.271, 41.056)	0.347	6.130	1.000
			Rarely	9.729 (0.987, 95.867)	0.051	18.945	1.000
			Never	26.249 (3.309, 208.252)	0.002	51.993	6.072
		Adjusted	Several times a month	2.420 (0.130, 45.136)	0.554	4.273	1.000
			Rarely	3.889 (0.284, 53.309)	0.309	7.241	1.000
			Never	15.893 (1.253, 201.504)	0.033	31.277	1.817
	AI-enabled triage guidance	Crude	Several times a month	0.106 (0.009, 1.218)	0.072	18.411	1.000
			Rarely	1.306 (0.270, 6.311)	0.739	1.939	1.000
			Never	1.501 (0.336, 6.713)	0.595	2.368	1.000
		Adjusted	Several times a month	0.149 (0.009, 2.394)	0.179	12.907	1.000
			Rarely	1.037 (0.187, 5.759)	0.967	1.232	1.000
			Never	1.112 (0.233, 5.312)	0.894	1.466	1.000
	AI-assisted robotic surgery	Crude	Several times a month	8.846 (0.682, 114.707)	0.095	17.176	1.000
			Rarely	17.612 (1.876, 165.364)	0.012	34.716	3.157
			Never	41.509 (5.191, 331.900)	0.000	82.514	9.856
		Adjusted	Several times a month	37.531 (0.631, 2232.708)	0.082	74.558	1.000
			Rarely	29.043 (0.852, 990.229)	0.061	57.582	1.000
			Never	61.031 (2.139, 1741.443)	0.016	121.559	3.700

Supplemental analysis of effect modification

The supplemental analysis of effect modification is shown in Table 5. For AI-assisted imaging interpretation, the interaction estimate had a confidence interval that did not include 1.0, but the interval was very wide. For the other outcomes, the confidence intervals included 1.0. Overall, the interaction analysis did not provide precise evidence of heterogeneity by occupational subgroup.

**Table 5:** Multiplicative interaction between generative AI use group and occupation for non-acceptance of each medical AI application Data are presented as exponentiated interaction-term coefficients with 95% confidence intervals from models including GAI use group, occupation, and their product term. These estimates represent ratios of PRs on the multiplicative scale for non-acceptance of each medical AI application; specifically, they indicate how the association between the lower-use group (vs the higher-use group) and non-acceptance differed between non-healthcare workers and healthcare workers. Values greater than 1 indicate a stronger association among non-healthcare workers than among healthcare workers, whereas values less than 1 indicate a stronger association among healthcare workers. Crude and adjusted estimates were obtained using modified Poisson regression with robust standard errors. p-values were derived from Wald tests and were not adjusted for multiple comparisons. A p-value of <0.05 was regarded as nominal statistical support. PR, prevalence ratio; aPR, adjusted prevalence ratio; CI, confidence interval; AI, artificial intelligence; GAI, generative artificial intelligence

Outcome	Model	Interaction estimate (95% CI)	p-value
AI-assisted imaging interpretation	Crude	12.441 (1.164, 133.008)	0.037
	Adjusted	13.960 (1.075, 181.342)	0.044
AI-based health risk prediction	Crude	0.801 (0.099, 6.450)	0.835
	Adjusted	0.945 (0.105, 8.469)	0.960
AI-based treatment recommendations	Crude	2.337 (0.276, 19.826)	0.436
	Adjusted	2.311 (0.262, 20.403)	0.451
AI-enabled triage guidance	Crude	0.621 (0.088, 4.401)	0.633
	Adjusted	0.693 (0.150, 3.202)	0.638
AI-assisted robotic surgery	Crude	2.663 (0.396, 17.930)	0.314
	Adjusted	3.089 (0.455, 20.992)	0.249

## Discussion

Key results

This exploratory cross-sectional web survey examined whether routine GAI use was associated with acceptance of five medical AI application scenarios, stratified by occupation. In adjusted analyses, the lower-use group had a higher prevalence of non-acceptance of AI-based health risk prediction and AI-based treatment recommendations among healthcare workers. Among non-healthcare workers, this pattern was also observed for AI-assisted imaging interpretation and AI-assisted robotic surgery, whereas the association for AI-enabled triage guidance was imprecise in both strata. Taken together, these findings suggest that more frequent GAI use may be associated with greater acceptance of selected medical AI applications, and that this pattern may differ across application domains and occupational contexts. However, several estimates had wide confidence intervals, and these results should be interpreted as imprecise and exploratory rather than as evidence of large or stable effects.

The sensitivity analysis using four-level GAI use frequency yielded findings broadly consistent with the primary dichotomous analysis. Although the estimates were imprecise, the direction of association generally suggested greater non-acceptance of medical AI among participants with less frequent GAI use. This supports, but does not confirm, the robustness of the primary findings.

A supplemental analysis using a product term provided statistical support only for AI-assisted imaging interpretation, and the confidence interval was extremely wide. Given the consideration of multiple outcomes, this interaction finding should be treated as hypothesis-generating rather than confirmatory.

Calibration weighting (raking) was used to align measured sociodemographic margins with external benchmarks. Therefore, the reported aPRs should be interpreted as weighted associations after adjustment to these measured margins, rather than as fully population-representative estimates for all Japanese internet users [16].

Interpretation

Because this was a cross-sectional study, the following interpretations should be viewed as hypothesis-generating rather than causal. Frequent GAI use may be a marker of broader technology-related orientation, such as digital confidence, openness to innovation, novelty enthusiasm, prior AI-related experience, or lower perceived uncertainty about algorithmic systems. Therefore, the observed associations should not be interpreted as evidence that routine GAI use caused greater acceptance of medical AI.

Among healthcare workers, associations were supported for domains proximal to clinical reasoning and decision-making (risk prediction and treatment recommendations). This pattern is consistent with the hypothesis that individuals who routinely use GAI may be more familiar with AI-assisted information processing and may have lower psychological barriers to algorithm-supported decision-making. However, this pattern may also reflect general openness to technology, digital confidence, prior AI-related training, or occupational environments in which digital tools are more familiar. This interpretation is consistent with emerging evidence that exposure to ChatGPT is associated with shifts toward more favorable perceptions of AI in healthcare [9]. In addition, recent surveys emphasized the need for AI education for healthcare workers to facilitate appropriate acceptance of medical AI [10,21].

Among non-healthcare workers, associations were supported across more domains. One interpretation is that non-professional evaluations may rely more on general technology optimism, perceived convenience, and broad trust in digital systems, with less domain-specific differentiation based on accountability and professional norms. Public-opinion work also suggests that “trust” in healthcare AI is multidimensional and can vary by decision type and perceived bias or responsibility [2,22]. Therefore, broader acceptance among non-healthcare workers may reflect a more general attitudinal framework rather than domain-specific judgments.

The imprecise findings for AI-enabled triage guidance may indicate that prioritization decisions evoke stronger concerns about fairness, downstream harms, and accountability than other applications, thereby weakening the association with routine GAI use [23]. Alternatively, the scenario wording and dichotomization rule may have provided limited discrimination for this domain.

The imaging-specific interaction should be interpreted cautiously. Imaging AI is a comparatively mature and publicly visible application area, particularly within radiology workflows, and professional expectations and concerns in radiology have been documented in large surveys [24-26]. If occupation truly modifies the association with imaging AI, one hypothesis is that healthcare workers may evaluate it through a more domain-specific lens (workflow integration, liability, and professional identity), whereas non-healthcare workers may treat it as an archetypal “successful medical AI” use case. However, this hypothesis requires replication and more granular measurement of domain-specific knowledge and trust.

Comparison with prior work supports two broader implications. First, acceptance can differ between healthcare professionals and the public within the same national context, and the occupational context should therefore be treated as a central dimension when interpreting acceptance data [27]. Second, prior exposure to widely used GAI tools may be associated with differences in perceptions of healthcare AI over time, although the present cross-sectional design cannot determine whether GAI use precedes or changes medical AI acceptance [9]. These points motivate repeated surveys and longitudinal designs to clarify temporal ordering and identify mediators, such as trust in medical AI, perceived risk related to medical AI, digital confidence, and prior AI-related experience.

Practical implications

These findings may help health systems plan communication and education before introducing medical AI. Prior GAI use and occupation may be useful for identifying groups that differ in their acceptance of medical AI. Therefore, health systems should not assume that the same explanation or training will be appropriate for all stakeholders. Instead, they could first assess stakeholders’ prior AI experience, acceptance, concerns, and information needs, and then tailor clinician training or public communication accordingly.

Strength and limitations

This study has several strengths. Acceptance was assessed across five distinct application scenarios rather than as a single global attitude, allowing domain-specific patterns to be explored. Stratified analyses by occupation aligned with prior evidence that acceptance differs between healthcare professionals and the public [27]. In addition, calibration weighting was used to improve alignment with measured external sociodemographic margins for Japanese internet users [16].

Several limitations warrant attention. First, the cross-sectional design prevents establishing temporal ordering; reverse causation cannot be excluded.

Second, exposure and outcomes were self-reported and dichotomized, potentially leading to misclassification and information loss. In addition, acceptance of medical AI was measured using investigator-developed single-item measures for each scenario. These items were intended to capture stated acceptance in hypothetical scenarios and were not based on a validated psychometric scale. The questionnaire was also not pretested before fielding; therefore, no pilot survey or cognitive interviewing was conducted to confirm whether respondents interpreted the items on GAI use and medical AI acceptance as intended. This is particularly relevant because both GAI use and medical AI acceptance are relatively novel constructs, and respondents may have differed in how they understood these terms or the hypothetical clinical scenarios. Therefore, construct validity and reliability could not be formally assessed. Responses may also have been influenced by social desirability bias, and stated acceptance may differ from actual acceptance or behavior in real clinical settings. Such measurement error or misinterpretation could have attenuated or distorted the observed associations. Future studies should develop and validate measurement instruments for these constructs, including cognitive interviewing, pilot testing, and assessment of reliability and construct validity.

Third, the study used a non-probability commercial web-survey panel with first-come, first-served enrollment until occupation-specific quotas were reached. This sampling procedure may have introduced self-selection bias, digital literacy bias, and volunteer bias. In particular, individuals who were registered web-panel members and responded before the quotas were filled may differ from the broader population of Japanese internet users. Because GAI use is a technology-related exposure, participants with greater digital engagement, greater comfort with online tools, or stronger interest in new technologies may have been overrepresented. Such a selection could have inflated the observed associations between GAI use and acceptance of medical AI. Although calibration weighting was used to align the sample with external sociodemographic margins, weighting cannot fully eliminate selection bias arising from unmeasured differences between participants and nonparticipants. Conditioning on participation can distort associations when participation is related to both exposure and outcome [14]. Therefore, transparent reporting for web surveys is essential [12], and this paper has been reported in accordance with the guidelines.

Fourth, no power-based sample size calculation was performed because this study was exploratory and constrained by budgetary and operational considerations. The total sample size was 200, with 100 healthcare workers and 100 non-healthcare workers. Therefore, estimates from subgroup-specific and scenario-specific models may be unstable, particularly when the number of non-acceptance events is small. The extremely wide confidence intervals observed in some analyses should be interpreted as reflecting limited precision rather than strong or stable associations. In addition, for several aPDs, standard errors and confidence intervals could not be estimated because of sparse non-acceptance events. These absolute-scale estimates were therefore presented descriptively and should be interpreted with caution.

Fifth, multiple outcomes, occupational subgroups, and model specifications were examined, which increases the risk of false-positive findings. Although p-values were reported to provide nominal statistical support for exploratory interpretation, they were not adjusted for multiple comparisons and should not be interpreted as confirmatory evidence [15]. Therefore, isolated statistically supported findings should be interpreted cautiously, and the results require confirmation in future studies with prespecified hypotheses and adequate sample sizes.

Finally, residual confounding may persist because the number of measurable covariates was limited by budgetary constraints. Covariate selection followed causal principles to reduce substantial potential confounding [13], but unmeasured factors such as health literacy, risk sensitivity, digital literacy, digital confidence, novelty enthusiasm, broader openness to innovation, or prior AI experiences may still influence results. Although age and educational attainment were included as measured covariates, these variables may not fully capture generational differences in technology use or general technology orientation. In addition, occupation-specific confounders were not fully measured. Among healthcare workers, factors such as job role, clinical specialty, clinical experience, occupational culture, institutional exposure to medical AI, and prior AI-related training may affect both GAI use and acceptance of medical AI. Among non-healthcare workers, industry type, work-related digital tool use, and prior exposure to AI-enabled services may also influence the association. Therefore, the observed associations may partly reflect general technology orientation, digital confidence, novelty enthusiasm, or occupational norms rather than a specific causal role of GAI use, and residual confounding by occupation-specific factors cannot be excluded.

Generalizability and transportability

The target population was Japanese internet users. Nevertheless, the registered web panel and occupation-specific quotas limit generalization to the broader Japanese population. Calibration weighting improves alignment with respect to measured margins but cannot guarantee transportability to populations that differ in unmeasured determinants of both GAI use and acceptance [16]. Because technology adoption, trust in healthcare institutions, expectations about professional authority, and public narratives around AI may differ across countries, the findings should not be assumed to apply directly to healthcare systems outside Japan. Moreover, GAI use and acceptance of medical AI are likely to change rapidly as technologies, governance, and public narratives evolve. Therefore, replication across time points and settings is needed to evaluate the stability and transportability of the observed patterns.

## Conclusions

In this exploratory cross-sectional web survey of Japanese internet users, more frequent GAI use was associated with higher acceptance of selected medical AI applications, with patterns that differed between healthcare and non-healthcare respondents. The findings do not support causality, but they suggest that implementation strategies for medical AI may benefit from considering stakeholders’ prior experience with GAI and tailoring communication and training by occupational context. Future research should prioritize longitudinal designs and repeated surveys to clarify temporal ordering and to identify mediating factors such as trust and risk sensitivity.

## Appendices

Appendix 1

**Table 6:** English translation of the original Japanese web-based questionnaire used in the survey This table presents the English translation of the original Japanese web-based questionnaire. The questionnaire was administered in Japanese, and the English wording is provided for reporting purposes only. The table includes the screening questions, informed consent screen, five scenario-based items assessing acceptance of medical AI, and participant characteristic items. For the acceptance items (Q1-Q5), respondents selected one of four options: acceptable, somewhat acceptable, somewhat unacceptable, or unacceptable. Q12 allowed multiple responses. Participants who selected “Student” in S1, “Less than 3 years” in S2, or “Yes” in S3 were not eligible for the main survey. AI, artificial intelligence; CT, computed tomography; JPY, Japanese yen

Section	Item	Content presented to respondents	Response options/notes
Screening questions	S1	Please select the option that best describes your current occupation.	Healthcare worker (e.g., physician, nurse, pharmacist, other healthcare professional, or healthcare administrative staff); Occupation other than the above, Student; Note: Participants who selected “Student” were not eligible for the main survey.
Screening questions	S2	How many years of experience do you have in your current occupation?	Less than 3 years, 3 years or more; Note: Participants who selected “Less than 3 years” were not eligible for the main survey.
Screening questions	S3	Is your occupation directly related to the development of generative artificial intelligence, such as ChatGPT?	Yes No; Note: Participants who selected “Yes” were not eligible for the main survey.
Informed consent screen	Study invitation	Request for participation in the study	You are invited to participate in the study entitled “The impact of generative AI use on the acceptance of medical AI: a comparison of attitudes across application domains between healthcare workers and the general public,” conducted by Toshiharu Mitsuhashi, New Medical Research and Development Center, Institute of Medicine, Dentistry, and Pharmaceutical Sciences, Okayama University. Please read the study information carefully. Only those who agree to participate should proceed to the questionnaire.
Informed consent screen	Consent confirmation	I have read and understood the above information and agree to participate in this study.	Checkbox option: I agree
Informed consent screen	Start button	Button label	Start survey; Note: The button is enabled when the respondent ticks “I agree.”
Part 1. Acceptance of medical AI	Section instruction	The following section presents five different examples of medical AI. After reading each example, please indicate, assuming that you were the patient, to what extent you would accept the use of such AI in your own diagnosis or treatment. Please select one response for each item.	Applies to Q1-Q5
Part 1. Acceptance of medical AI	Q1	AI-assisted imaging interpretation AI analyzes your X-ray or CT images taken by a physician, identifies areas that may suggest disease such as cancer or fracture, and reports them to the physician. The final diagnosis is made by the physician with reference to the AI output. If you were the patient, to what extent would you accept the use of this AI in your own diagnosis?	Acceptable Somewhat acceptable Somewhat unacceptable Unacceptable
Part 1. Acceptance of medical AI	Q2	AI-based health risk prediction AI analyzes daily health data measured by devices such as a smartwatch that you wear, including heart rate, and predicts disease risk, for example, by warning that there is a high possibility of a dangerous arrhythmia occurring in the next few hours. The warning is provided to you and/or your family. If you were the patient, to what extent would you accept the use of this AI in your own health management?	Acceptable Somewhat acceptable Somewhat unacceptable Unacceptable
Part 1. Acceptance of medical AI	Q3	AI-based treatment recommendations AI comprehensively analyzes your lifestyle, past medical history, and the latest medical literature, and suggests to the physician several treatment options that may be most effective for you personally, such as types of medication and how they should be used. The final decision on which treatment to choose is made through discussion between you and your physician. If you were the patient, to what extent would you accept the use of this AI in decisions about your own treatment plan?	Acceptable Somewhat acceptable Somewhat unacceptable Unacceptable
Part 1. Acceptance of medical AI	Q4	AI-enabled triage guidance You interact with an AI chatbot on a hospital website. Based on the symptoms you enter, the AI asks additional questions, organizes the information, and advises you on the appropriate level of urgency and where to seek care, for example, “You should go to the emergency department” or “Please make an appointment with internal medicine.” If you were the patient, to what extent would you accept the use of this AI in deciding your own care-seeking behavior?	Acceptable Somewhat acceptable Somewhat unacceptable Unacceptable
Part 1. Acceptance of medical AI	Q5	AI-assisted robotic surgery When you undergo surgery, the physician operates a surgical support robot equipped with AI. The AI assists by correcting hand tremor and helping the physician stay within the preplanned surgical range, thereby enabling precise maneuvers that would be difficult for humans alone. The overall surgery is performed and supervised by a human physician. If you were the patient, to what extent would you accept the use of this AI in your own surgery?	Acceptable Somewhat acceptable Somewhat unacceptable Unacceptable
Part 2. About yourself	Section instruction	Next, we would like to ask about your own experiences and opinions.	Applies to Q6-Q12
Part 2. About yourself	Q6	Do you currently have a chronic disease that requires regular medical visits or treatment, such as hypertension, diabetes, or asthma?	Yes No
Part 2. About yourself	Q7	How often do you use generative AI in daily life outside of work, such as AI that generates text or images, for example ChatGPT?	Almost every day Several times a week Several times a month Hardly ever
Part 2. About yourself	Q8	How often do you use generative AI for work, including study?	Almost every day Several times a week Several times a month Hardly ever
Part 2. About yourself	Q9	What is your highest level of education?	Junior high school, High school, Vocational school or junior college, University, Graduate school
Part 2. About yourself	Q10	Please select the category closest to your household’s total annual income before tax in the previous year.	Less than JPY 3 million JPY 3 million to less than JPY 5 million JPY 5 million to less than JPY 8 million JPY 8 million to less than JPY 12 million JPY 12 million or more Prefer not to answer
Part 2. About yourself	Q11	In general, how interested are you in information about technology, such as new smartphones, applications, or web-based services?	Very interested Somewhat interested Neither interested nor uninterested Not very interested Not interested at all
Part 2. About yourself	Q12	Regarding medical AI in general, please select all items that apply to the points you expect from it and the points that concern you or make you feel uneasy.	Expected benefits: Improved diagnostic accuracy, Shorter waiting times, Reduced medical costs, Reduced workload for physicians and nurses, Development of new treatments, Other (please specify). Concerns: Risk of misdiagnosis, Leakage of personal information, Reduced communication with physicians, Unclear responsibility, Standardization of care or loss of individualized care, Other (please specify)
Final screen	Completion message	Thank you very much for your participation. This completes the survey.	End of questionnaire
